# Pulmonary Complications of Everolimus in Liver Transplant Patients: A 10-Year Experience

**DOI:** 10.7759/cureus.53334

**Published:** 2024-01-31

**Authors:** Mark S Obri, Alan M Fahoury, Suhaib Alhaj Ali, Momin Samad, Spandana Alluri, Alex S Obri, Mohamed Ramzi Almajed, Kevin B Harris, Syed-Mohammed Jafri

**Affiliations:** 1 Internal Medicine, Henry Ford Health System, Detroit, USA; 2 Internal Medicine, University of Toledo College of Medicine and Life Sciences, Toledo, USA; 3 Pharmacy, College of Pharmacy, University of Toledo, Toledo, USA; 4 Gastroenterology, Henry Ford Health System, Detroit, USA; 5 Gastroenterology and Hepatology, Henry Ford Health System, Detroit, USA

**Keywords:** pulmonary hypertension, pulmonary interstitial fibrosis, copd, calcineurin inhibitors, everolimus toxicity, liver transplant

## Abstract

This retrospective study aims to evaluate the safety of everolimus when used as part of the immunosuppression regimen in patients who underwent liver transplant from 2009 to 2019 at a tertiary liver transplant center. Patients were divided into two groups: those who received everolimus as part of the post-transplant regimen and those who did not. The primary safety outcome measured was the development of new pulmonary complications that had been associated with everolimus use in prior studies. Lung function was determined by pulmonary function tests if available or CT scans of the chest. Secondary outcomes measured included everolimus discontinuation rates and survival rates. During the study period, 450 patients underwent liver transplant; 35% of patients received everolimus (n=156) and 65% of patients did not receive everolimus (n=292). Primary safety outcome of pulmonary complications was seen in 3.9% of patients who received everolimus (n=6) and 6.3% of the control group patients who did not receive everolimus (n=19). The association between everolimus use and new pulmonary complications was not significant with a chi-square statistic of 1.33 (p=0.249). Overall, 51.3% of patients who received everolimus during their post-transplant course discontinued the medication (n=80). Everolimus is safe from a pulmonary toxicity standpoint in liver transplant immunosuppression regimens as there was no significant difference found in pulmonary complications between patients who received the medication and those who did not.

## Introduction

Immunosuppressive therapy is an essential component of the patient’s course after liver transplantation. Developments and research into the optimal regimen have led to better patient outcomes and has decreased graft rejection rates. Common post-transplant therapies use a combination of immunosuppressant agents, such as calcineurin inhibitors and mammalian target of rapamycin (mTOR) inhibitors [1]. Some examples of these medications include cyclosporine A, tacrolimus, and everolimus [1]. mTOR inhibitors act by halting the cell cycle from progressing into the S-phase by binding the FK506-binding protein 12, which leads to G1 cell cycle arrest and, consequently, inhibition of T-cell mediated immunity [2]. Everolimus is an mTOR inhibitor that is commonly used in liver transplant immunosuppression regimens [3].

Although everolimus is an effective therapy after solid organ transplant, it still comes with concerning adverse effects. Common medication toxicities include fatigue, stomatitis, leukopenia, and anemia [4]. Other toxicities include nausea, vomiting, diarrhea, thrombocytopenia, and dyslipidemia [2,4]. Several publications have reported the development of pulmonary complications, such as interstitial pneumonitis, after solid organ transplants; in these patients, the pulmonary complications were noted to improve after discontinuation of everolimus [5-7]. While the mechanism of everolimus-associated pulmonary complications is not understood, some investigators have speculated that mTOR inhibition is associated with activation of the *STAT1* gene, leading to increased apoptosis and injury; others theorize that a *STAT3* polymorphism may increase the risk of interstitial lung disease with mTOR inhibitors [8,9]. Moreover, studies have also indicated that pulmonary toxicity seen with mTOR inhibitors correlated with higher doses of the medication [10].

Prior to prescribing these medications, physicians and patients need to be informed about the potential toxicities as these adverse effects can dictate immunosuppressive regimens. We aim to evaluate the safety of everolimus when used as a component of the immunosuppression regimen in liver transplant patients.

## Materials and methods

Methods

A single-center retrospective study was conducted at a tertiary liver transplant center. All patients who underwent liver transplant from 2009 to 2019 were included. Patients were divided into two groups: those who received everolimus as part of the post-transplant immunosuppressant regimen and those who did not. The primary safety outcome measured was the development of new pulmonary complications. Pulmonary complications were limited to those that had been associated with everolimus use in prior studies; these include chronic obstructive pulmonary disease, pulmonary interstitial fibrosis, and pulmonary hypertension. Complications were assessed on pulmonary function tests (PFTs) and computed tomography (CT) scan of the chest.

Pulmonary complications were assessed on PFTs by comparing PFTs performed before and after transplant. CT scan of the chest was used to evaluate for radiographic pulmonary changes; formal radiology interpretations were used to determine whether changes were abnormal or new. Charts were reviewed for documentation by the patients’ transplant team regarding the development of new pulmonary symptoms and conditions.

Secondary outcomes

Secondary outcomes measured included the rates of everolimus discontinuation and survival. Patients’ medical charts were reviewed for documentation regarding the reason for everolimus discontinuation. Patients were excluded if the medication was discontinued and the documentation did not report the underlying reason for discontinuation. Survival rate was assessed based on whether the patient had died at the time of last known follow-up. Patients were excluded if they did not have a follow-up of at least one month.

Data and statistics

Data of patients who underwent a liver transplant from 2009 to 2019 at a tertiary liver transplant center were extracted from an electronic medical record. Charts were individually reviewed for documentation, care regimen, imaging, medications, and complications. A chi-square analysis was conducted using a statistical analysis program to evaluate the relationship between occurrence of the primary outcome in the group that received everolimus and the group that did not. It was also used to determine if there was a statistically significant difference in the overall survival between the two groups. Results were considered statistically significant if the p-value was less than 0.05.

## Results

A total of 450 patients underwent liver transplant during the study period; 64% of the patients were men (n=288) and 83.6% of the patients were white (n=376). Overall, 35% of patients received everolimus (n=156) and 65% of patients did not receive everolimus (n=292) (Table 1).

**Table 1:** Overall liver transplant patients who received everolimus and those who did not

	Yes	No
Received Everolimus	35% (n=156)	65% (n=292)

Rates of pulmonary toxicity

Primary safety outcome of pulmonary complications was seen in 3.9% of patients who received everolimus (n=6) and 6.3% of the control group patients who did not receive everolimus (n=19). Among patients who did not receive everolimus, patient pulmonary problems developed were chronic obstructive pulmonary disease (COPD) 42.1% (n=8), pulmonary fibrosis 42.1% (n=8), and asthma 15.9% (n=3). Among patients who received everolimus, pulmonary complications included COPD in 66.7% of patients (n=4), pulmonary fibrosis in 16.7% of patients (n=1), and asthma in 16.7% of patients (n=1). The association between everolimus use and new pulmonary complications was not statistically significant with a chi-square statistic of 1.33 (p=0.249), which was not significant at p < 0.05 (Table 2).

**Table 2:** New pulmonary complications in liver transplant patients who received everolimus compared to those who did not

	Received Everolimus	Control group	p-Value (p < 0.05)
Overall new pulmonary complications	3.9% (n=6)	6.3% (n=19)	0.249
Chronic obstructive pulmonary disease	66.7% (n=4)	42.1% (n=8)	-
Pulmonary fibrosis	16.7% (n=1)	42.1% (n=8)	-
Asthma	16.7% (n=1)	15.9% (n=3)	-

Follow-up

Patients started everolimus on an average 208 days after transplant (range: 35-2,261 days), and the mean last known follow-up after everolimus initiation was 50.3 months. Patients who received everolimus had a mean follow-up of 57.6 months, whereas those who did not had a mean follow-up of 48.6 months.

Rates of discontinuation

Overall, 51.3% of patients who received everolimus during their post-transplant course discontinued the medication (n=80). Reason for everolimus discontinuation was mainly non-pulmonary adverse events including proteinuria, oral ulcers, and cost. In addition, 76.3% of the patients had multiple reasons for discontinuation (n=61). Review of liver transplant documentation did not reveal any patients in whom pulmonary toxicity was attributed as the cause of everolimus discontinuation.

Everolimus and survival

Of patients who received everolimus, 79.3% were alive at their last known follow-up (n=80), whereas 84.0% of patients who did not receive everolimus were alive at their last known follow-up (n=245). When comparing the survival rates at the time of mean follow-up, the difference was not statistically significant with a chi-square statistic of 1.41 (p=0.24), which was not significant at p < 0.05.

## Discussion

The aim of this study was to evaluate whether an association exists between everolimus and pulmonary toxicity. While previous studies have shown that mTOR inhibitors can cause interstitial pneumonitis, we also included COPD and pulmonary hypertension due to the theoretical risk that other pulmonary toxicity may be present. Our data show that the occurrence of new pulmonary complications after liver transplant in the group that took everolimus was comparable to the group that did not take it. Furthermore, to our knowledge, there have only been four reported cases in the literature of interstitial pneumonitis after liver transplant [5,11-13]. Therefore, in the absence of data suggesting a correlation between everolimus and pulmonary complications, everolimus appears to have an acceptable pulmonary safety profile when used as part of the immunosuppressive regimen in liver transplant patients.

Current data have shown numerous instances of pulmonary toxicity in patients taking everolimus after solid organ transplants, which, in many cases, was noted to resolve after discontinuation of the medication. In the majority of reported cases, the main adverse pulmonary complication was interstitial pneumonitis. Patients in these cases typically presented clinically with symptoms of fever, hypoxia, dyspnea, cough, and generalized weakness after using everolimus for weeks to months [5-7,14]. Subsequently, chest imaging with X-ray and CT scans showed varying pulmonary infiltrates in these patients [5,6,14]. Interestingly, plasma serum levels of everolimus are suspected to play a role in pulmonary toxicity in solid organ transplant, and several of the reported liver transplant serum levels were either slightly below or within therapeutic levels of 5.0 to 12.0 ng/mL [5,10-12]. In the reported liver transplant cases, everolimus was eventually discontinued and patients were treated with or without corticosteroids.

We do note a 51.3% (n=80) discontinuation of everolimus in our patient population, which complicates our evaluation of the drug’s safety. Our analysis showed that the most common independent adverse effects leading to discontinuation were oral ulcers and proteinuria, with cost of the medication also playing a major role. Stomatitis is a well-documented adverse effect of everolimus, with a meta-analysis showing that approximately 43% of cases analyzed reported an occurrence [4]. While the same analysis does not mention a significant incidence of proteinuria, there are a plethora of documented cases of proteinuria after everolimus usage [15-17]. Additionally, our results also indicated that approximately 76.3% of patients discontinued everolimus due to multiple reasons (n=61). From the same meta-analysis, the second and third most common side effects of everolimus were leukopenia and anorexia [4]. Other notable adverse effects that could have been reasons for discontinuation were fatigue, nausea, vomiting, anemia, thrombocytopenia, and dyslipidemia [3,4].

Our study is limited due to its retrospective nature, the non-uniformity of repeat CT imaging, and our reliance on subjective documentation. Objectively, we compare CT scans for imaging of the lungs, but for any pulmonary symptoms or side effects, we rely on the documentation of the hepatologist, which is not always uniform. We also noted that patients often discontinued everolimus for one or multiple reasons, and the reason for discontinuation is not always clearly documented and often is generally labeled as “side effects.” Regardless, the percentage of discontinuation offers valuable data on the tolerability of the medication and insight for prescribers.

## Conclusions

Everolimus is a safe component of the liver transplant immunosuppressant regimen from a pulmonary toxicity standpoint as no significant difference was found in terms of pulmonary complications between patients who received the medication and those who did not. Our study is limited due to a high rate of medication discontinuation not related to the outcome measures. Further large longitudinal multicenter studies are necessary to evaluate the safety and cost of everolimus and assess the correlation and causation between the medication and pulmonary complications.
